# Preoperative Use of a Mobile Application Within the Multidisciplinary Team Approach – A Randomized Controlled Clinical Trial

**DOI:** 10.1007/s11695-026-08768-1

**Published:** 2026-06-15

**Authors:** Celine Kadesch, Christel Weiss, Michael Hetjens, Niki Taebi, Sebastian Schölch, Christoph Reissfelder, Susanne Blank, Mirko Otto, Cui Yang

**Affiliations:** 1 Department of Surgery, University Medical Centre Mannheim, Mannheim, Germany; 2 Department of Biomedical Informatics, Mannheim Institute for intelligent Systems in Medicine, Medical Faculty Mannheim, Heidelberg University, Mannheim, Germany; 3 JCCU Translational Surgical Oncology (A430), German Cancer Research Center (DKFZ), Heidelberg, Germany; 4 DKFZ-Hector Cancer Institute, German Cancer Research Center, Heidelberg, Germany

**Keywords:** Perioperative management, Obesity, Mobile health application, eHealth, Bariatric surgery

## Abstract

**Background:**

Preoperative multidisciplinary team assessment (MTA) for bariatric surgery is complex and associated with high attrition rates. Digital health applications may improve patient engagement and completion rates, but evidence remains limited.

**Methods:**

In this prospective randomized controlled trial conducted at a tertiary bariatric center, patients aged ≥ 18 years with obesity II with obesity related medical problems or obesity III were randomized 1:1 to receive either standard multidisciplinary care alone (control group) or standard care plus a smartphone application (app group). The app provided digital checklists, appointment reminders, and automated alerts to healthcare providers for missed appointments. The primary outcome was successful completion of MTA, defined as being scheduled for metabolic and bariatric surgery or achieving sufficient weight loss, assessed at 18 months.

**Results:**

Among 223 enrolled participants, 178 (79.8%) completed follow-up (91 control, 87 app group). 115 participants (64.6%) successfully completed the MTA, with no significant difference between groups (62.6% control vs. 66.7% app group, *p* = 0.5741). However, post hoc stratification by engagement level revealed significant differences: participants with frequent app use (≥ 34% optimal app usage, *n* = 72) achieved 76.4% successful MTA completion versus 20.0% in low-engagement users (*n* = 15) and 62.6% in controls (*p* = 0.0002). Lower baseline BMI independently predicted successful MTA completion. Quality of life improved across all groups, while weight and body composition remained unchanged during the assessment period.

**Conclusion:**

A mobile health application can enhance completion rates of preoperative bariatric surgery assessment, but effectiveness is associated with user engagement. Digital health interventions should incorporate strategies to promote sustained engagement, with early identification and support for low-usage patients.

**Supplementary Information:**

The online version contains supplementary material available at 10.1007/s11695-026-08768-1.

## Key Points

Higher app engagement was associated with higher successful MTA completion rates.

An engagement threshold of 34% distinguished successful MTA completion.

Mobile health (mHealth) in bariatric surgery requires strategies to promote patient engagement.

**Supplementary Information:**

The online version contains supplementary material available at 10.1007/s11695-026-08768-1.

## Introduction

Obesity is a growing global health concern. Bariatric surgery has been shown to be the most successful solution against obesity, with patients continuing to lose weight even years after surgery [1]. Following metabolic and bariatric surgery (MBS), the prevalence of associated medical problems like diabetes, hypertension and sleep apnea also declines [2].

According to German guidelines, a patient must either present with obesity III or above or obesity II with at least one obesity related medical problem in order to be qualified for MBS [3]. General recommendations for patients prior to bariatric surgery are a series of preoperative assessments and interventions for evaluation and optimization of associated medical problems as well as weight loss within a multidisciplinary team approach (MTA). The MTA typically involves dietary counseling, psychological assessment, and evaluation of obesity-related medical conditions. Dietary counseling aims to establish individualized nutrition programs, correct micronutrient deficiencies, and adapt eating and lifestyle habits [4, 5]. Psychological assessment addresses the higher prevalence of psychiatric disorders, disordered eating, and psychosocial difficulties among bariatric candidates, and helps identify contraindications or conditions requiring stabilization prior to surgery [6, 7]. If the conservative treatment attempt as a combination of dietary education, behavioral therapy and regular physical exercise is not successful regarding optimal clinical response, the patients should be scheduled for surgery [3]. Because obesity related medical problems are frequently misdiagnosed prior to bariatric surgery, preoperative multidisciplinary evaluations for endocrine, gastrointestinal, cardiovascular and respiratory diseases are widely recommended to reduce risk for complications or mortality [8–12]. Moreover, previous studies have discovered an association between preoperative patient education with subsequent weight loss and lower mortality as well as higher weight reduction after surgery [13–15]. Thus, the completion of MTA is required before undergoing bariatric surgery in some European countries.

However, various barriers preventing patients from completion of the MTA have been identified previously [16], e.g. lack of understanding regarding bariatric surgery, scarcity of resources to facilitate collaborative decision-making regarding bariatric surgery, and unclear departmental boundaries (specialty versus primary care) etc. While the COVID-19 pandemic hastened the global adoption of mobile health (mHealth) in all health systems, mHealth was verified to have positive influences on quality of life, eating psychopathology, and behavioral patterns regarding food and physical activity in patients undergoing bariatric surgery [17–22]. Due to factors such as an increase in self-management and personalized feedback, mHealth is considered to be more efficient than standard care [23]. Beside optimizing preoperative preparation before some surgical interventions [24, 25], mHealth also allows to track complications und guarantee a continuous follow-up after surgery [17, 26]. However, evidence for usage of app assistance to complete MTA before bariatric surgery is lacking.

The goal of our study is to determine whether a smartphone application can increase the rate of successful MTA completion and subsequent metabolic bariatric surgery (MBS) within a multidisciplinary team approach.

## Methods

### Study Design

This study used a prospective, randomized design to investigate the feasibility and effectiveness of using a mobile application to support patients before bariatric surgery. Informed consent was obtained prior to enrollment in accordance with a clinical trial protocol approved by the local Ethical Committee. The study was conducted at a bariatric center of a tertiary care clinic and registered in the German Clinical Trials Register.

### Inclusion

Patients aged 18 years or older presenting in the bariatric center for their first consultation were screened consecutively. The inclusion criteria were: (1) obesity II with one or more associated medical problems (e.g., diabetes, arterial hypertension, sleep apnea) or obesity III and above; and (2) ownership of a smartphone with internet access and demonstrated ability to use it. Patients unable to provide written informed consent, those with cognitive impairments, or significant language barriers preventing understanding and consent in German (the language of the app) were excluded.

After the informed consent forms were signed, participants were randomized into standard group (non-app group) and intervention group (app group) using the Research Electronic Data Capture (REDCap) web application, stratified by sex, with an allocation ratio of 1:1. Stratification by sex was performed because women represent approximately 70–85% of MBS patients despite similar obesity prevalence across sexes [27–29], and male sex has been identified as a risk factor for preoperative dropout [30]. Blinding of participants and staff is not feasible due to the nature of the intervention. Upon enrolling in the study, we collected each patient’s demographics, obesity-associated medical conditions, past medical and surgical history, and conducted baseline assessments of height, weight, body impedance analysis (BIA), and Body Mass Index (BMI).

As this was an exploratory study without preliminary data to inform effect size estimates, a formal a priori power calculation was not feasible. We therefore pragmatically determined a target sample size of 200 patients (100 per group) based on feasibility considerations and the need to generate robust preliminary data for hypothesis generation.

### Standard Care in the Preoperative Phase and MTA

All participants were required to complete a comprehensive preoperative assessment provided by a multidisciplinary team. The assessment consisted of multiple specialist consultations for the evaluation and optimization of obesity-related medical conditions such as diabetes and obstructive sleep apnea; intensive nutritional counseling; exercise assessment; and psychological screening with appropriate intervention when necessary. This process typically required six months but could be shortened to three months for patients presenting with obesity IV or V, severe obesity related medical problems, or those for whom the multidisciplinary team deemed conservative treatment unlikely to succeed or futile.

After successful completion of the preoperative assessment, all cases were reviewed at a multidisciplinary conference. Once a consensus recommendation was reached, participants were informed and scheduled for surgical assessment in the outpatient clinic.

### Intervention

**Fig. 1:**
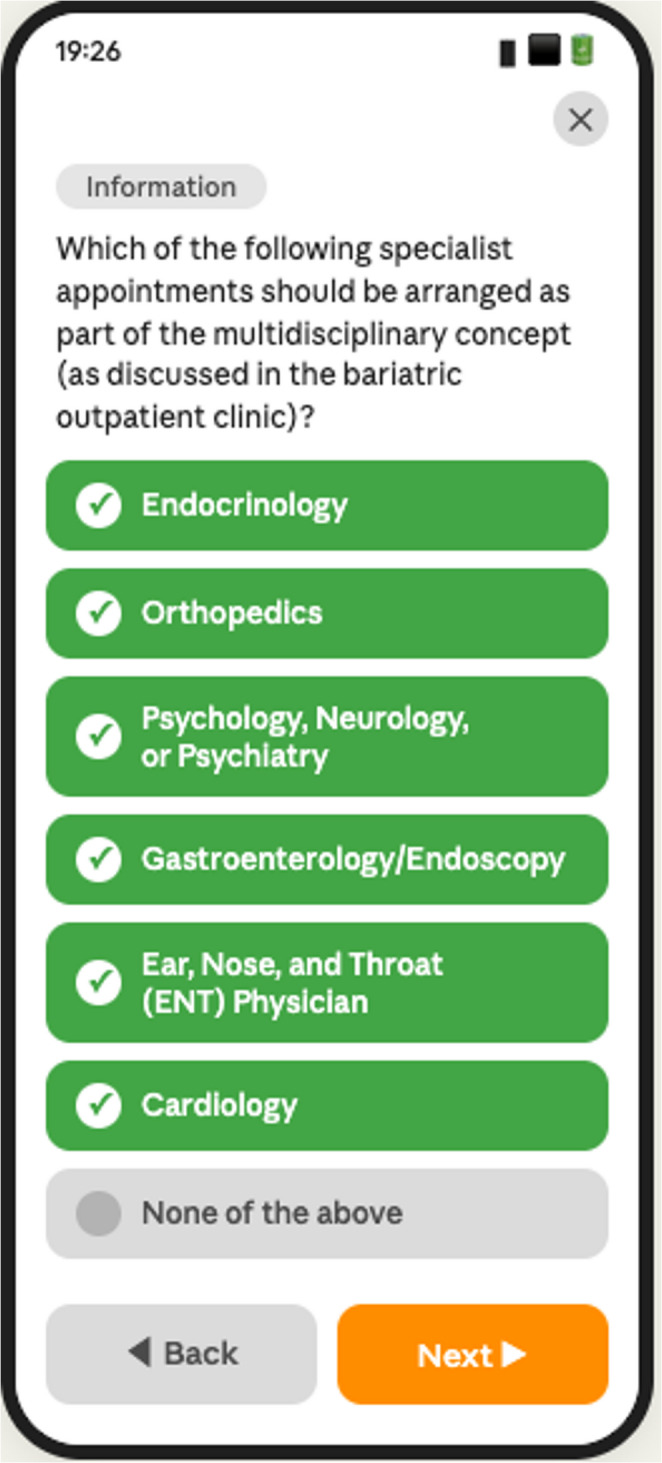
Screenshot of app showing question to check specialist appointments

The smartphone application App Care4Today^®^ Monitor (Johnson & Johnson Medical GmbH) was installed on participants’ personal smartphones after obtaining written informed consent. Participants were trained until they were confident with the app. The app guided participants through preoperative preparation and served as a digital checklist and reminder tool by presenting questionnaires regularly. The questionnaires comprised 19 items related to specialist appointments and four additional items assessing physical activity in a diary format (e.g., “Did you engage in physical activity today?” with answer options “Yes”, “No”, or “I am unable to exercise due to medical reasons (confirmed by a doctor’s note).”). The app was programmed to schedule question repetitions based on previous answers, providing customized reminders to each patient throughout the MTA process. Additional modules enabled self-monitoring of dietary caloric intake and body weight. (Fig. 1) An informational portal within the app provided direct access to the hospital website for evidence-based medical information. A list of questionnaire items is provided in Supplementary Table 1.

To better track progress, the app generated automated alerts when participants failed to complete required appointments within the specific timeframe. The alerts were forwarded to health care professionals by e-mail so that additional help could be offered. For example, if participants did not manage to schedule appointments with dieticians within three months of enrollment, the study team would proactively provide a list of dieticians in their local area.

### Assessment of Outcomes

For participants who completed the preoperative assessment and were scheduled for surgical evaluation in the outpatient clinic, the surgical assessment appointment marked the study endpoint. Participants who were not scheduled for surgical assessment within 18 months of enrollment (e.g., those who failed to complete the preoperative assessment, underwent surgery at another bariatric center, or achieved sufficient weight loss such that surgery was no longer indicated) were contacted by study personnel to assess study endpoints.

The primary outcome was successful completion of the preoperative assessment, defined as either: (1) being scheduled for bariatric surgery, or (2) achieving sufficient weight loss such that surgery was no longer clinically indicated. This study aimed to determine whether supplemental use of a mobile application during the preoperative assessment improved the rate of successful completion compared to standard care alone.

Demographic and clinical parameters were assessed at enrollment (baseline) and at the end of the study (at the time of surgical scheduling or at 18 months post-enrollment, whichever occurred first).

#### Weight Loss

The degree of weight loss achieved by participants before surgery measured in total weight loss in kilograms. The percentage of total weight loss (%TWL = weight at first visit – weight at last visit)/ weight at first visit] × 100) has been reported to be less influenced by confounding factors and was thus assessed [31]. 

#### BIA

Assessing changes in body composition with parameters resting metabolic rate, phase angle, total body water, lean body mass, extra cellular mass, body cell mass, % of BCM in Lean Body mass, Body Fat in Kilogram and % to monitor body changes in the MTA more strictly. BIA was assessed using the equipment Nutriguard-MS Body Impedance Analyzer (Data Input GmbH, Pöcking, Germany).

#### Quality of Life (QOL)

Measured using the Bariatric Quality Of Life Index (BQL) from the national registry, both at the start and end of the study. The BQL consists of 13 items with a Likert scale ranging from 1 to 5. Item scores are added for receiving the final score with a higher score correlating with a better quality of life.

#### Appointment Adherence

For each mandatory item of the MTA (nutritional counseling, sports diary, psychological consultation, endocrinology consultation, sleep apnea screening and upper endoscopy) as well as for additional appointments (pH-metry, cardiological consultation, orthopedic consultation) we monitored the completion of each appointment and calculated completion rates per group.

#### Assessment of Obesity-Related Medical Conditions

Obesity-related medical conditions such as arterial hypertension, type 2 diabetes mellitus, insulin resistance, obstructive sleep apnea and dislipidemia were documented at baseline (first visit) and last visit.

#### Barriers to Successful MTA Completion

For participants who failed to complete the MTA, barriers to completion were documented through telephone interviews conducted by study personnel. Participants were asked open-ended questions about obstacles.

### Statistical Methods

Statistical analysis was performed using SAS, release 9.4 (Statistical analysis system). For quantitative variables mean and standard deviation have been calculated. For qualitative factors, absolute and relative frequencies are given.

In order to compare the mean values of two groups, 2 sample t test has been performed. For skewed variables, a Wilcoxon 2 sample has been used instead.

Qualitative factors have been compared using a Chi^2^ test or Fisher’s exact test if the conditions of the Chi2 test were not fulfilled. In order to compare three groups, a one way ANOVA, a Kruskal-Walls test, Chi2 test or Fisher’s exact test has been conducted, as appropriate.

A logistic regression analysis with the binary outcome “successful completion of MTA” has been performed in order to find an optimal cut-off. Furthermore, a multiple logistic regression has been conducted in order to evaluate several factors and their impact on the outcome simultaneously.

In general, the result of a statistical test has been considered as statistically significant for p less than 0.05.

## Results

### Baseline Characteristics of Participants

**Fig. 2:**
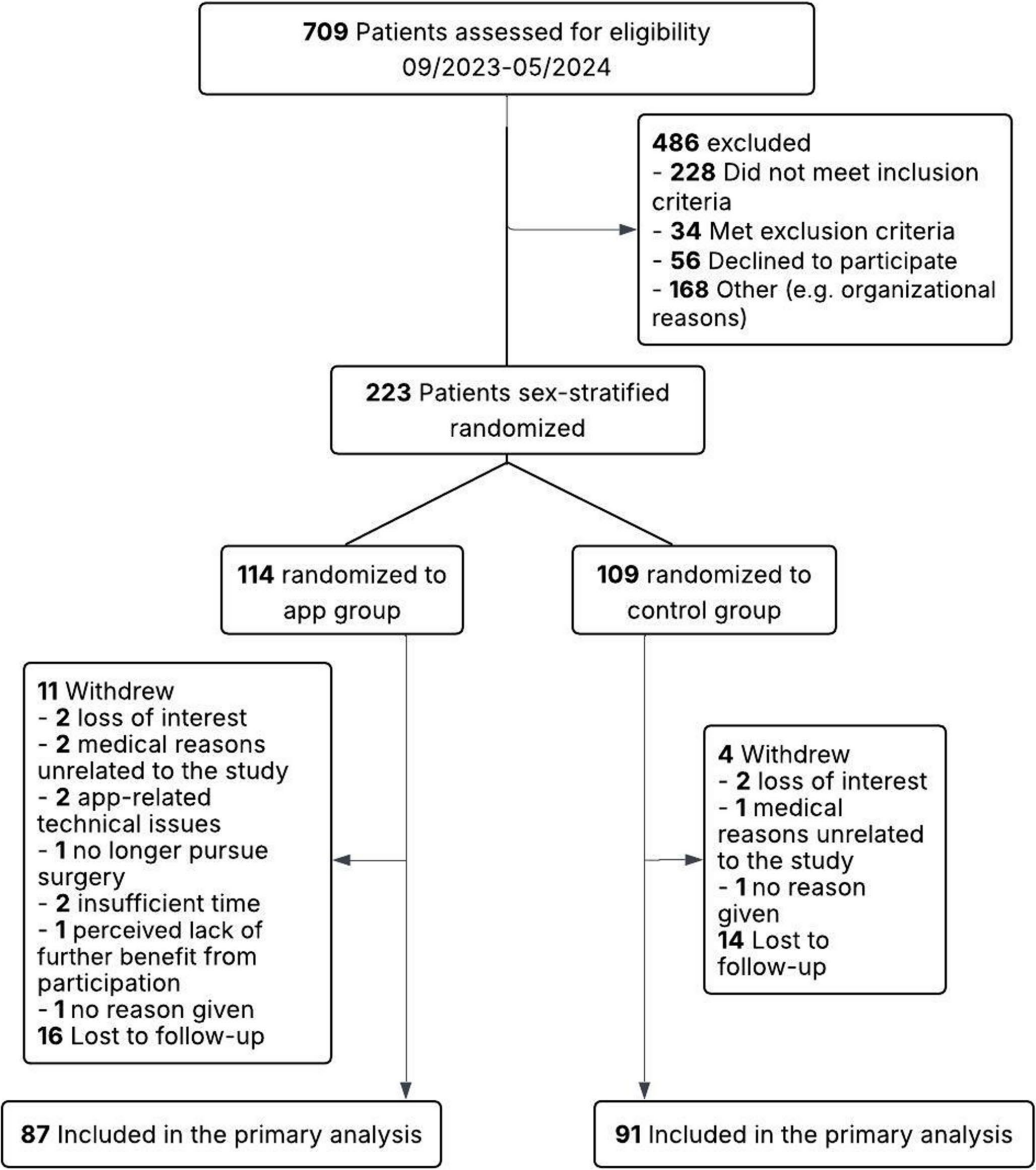
Flowchart of the trial

Between September 2023 and May 2024, 223 patients agreed to participate in the study. 45 patients (20.2%) were not available for outcome assessment (15 withdrew their consent, 30 were lost to follow-up). Reasons for withdrawal of consent were: loss of interest (*n* = 4), medical reasons unrelated to the study (*n* = 3), app-related technical issues (*n* = 2), decision to discontinue the MTA and no longer pursue surgery (*n* = 1), insufficient time for study participation (*n* = 2), no specific reason given (*n* = 2), and perceived lack of further benefit from participation (*n* = 1). Therefore, 178 participants (79.8%) were included in the final analysis, of which 91 (51.1%) were in the control group and 87 (48.9%) in the app (interventional) group (Fig. 2). Overall, 118 (66.3%) were female and 60 (33.7%) were male. Mean age was 40.3± 11 years (range 19–66), initial body weight 138.7 ± 24.8 kg (96–240) and initial BMI 46.5 ± 6.6 (35–72). Employment and education were comparable between groups. Most participants were employed full-time (54.5%), and 52.6% had completed vocational education. No patients in our cohort were excluded from surgery due to severe medical or psychological contraindications. See Table 1.

**Table 1:** Baseline characteristics

	Control (*n* = 91)	App (*n* = 87)	*p*-values
Age (years)	39.2 ± 10.9	41.5 ± 11.1	0.1495
Height (cm)	172.0 ± 10.1	171.1 ± 9.4	0.5584
Weight (kg)	141.3 ± 26.4	136.0 ± 22.8	0.1488
BMI (kg/m2)	47.2 ± 7.0	45.9 ± 6.1	0.1844
Female (n,%)	59 (64.84)	59 (67.82)	0.6741
MTA shortened to three months (n,%)	9 (9.9)	2 (2.3)	0.058
Education			0.2965
no education (n,%)	3 (3.5)	4 (4.7)	
secondary modern school (n,%)	26 (30.2)	28 (32.9)	
intermediate modern secondary school (n,%)	31 (36.1)	39 (45.9)	
university entrance qualification (n,%)	24 (27.9)	13 (15.3)	
others (n,%)	2 (2.3)	1 (1.2)	
Employment			0.5774
not employed (n,%)	7 (8.3)	12 (14.5)	
homemaker (n,%)	8 (9.5)	4 (4.8)	
incapable of working (n,%)	3 (3.6)	1 (1.2)	
retired (n,%)	3 (3.6)	2 (2.4)	
full time (n,%)	47 (56.0)	44 (53.0)	
part time 15–36 h per week (n,%)	13 (15.5)	13 (15.7)	
part time < 15 h per week (n,%)	1 (1.2)	4 (4.8)	
other (n,%)	2 (2.4)	3 (3.6)	

*BMI* body mass index. Qualitative factors are presented by their absolute and relative frequencies; for quantitative variables, mean ± standard deviation are given

### Primary Endpoint: Successful Completion of MTA

Overall, 115 participants (64.6%) completed the MTA successfully. The proportion of successful completion of MTA was slightly higher in the app group than in the control group (66.7% vs. 62.6%), but the difference was not statistically significant (*p* = 0.5741).

In a logistic regression restricted to this group, higher app use (percentage of optimal app use) was associated with successful completion of MTA (*p* = 0.0398). Optimal app use was defined as completion of all available checklists and interactions at each time point. An engagement level of 34% maximized sensitivity plus specificity (Supplementary Fig. 1). Using this cut-off, we split the app group into “app frequent (≥ 34% of optimal app use, n = 77) and “app seldom” (< 34% of optimal app use, n = 10). Baseline age, sex, weight, BMI, employment and education status did not differ across the three groups (all p > 0.05).

**Fig. 3:**
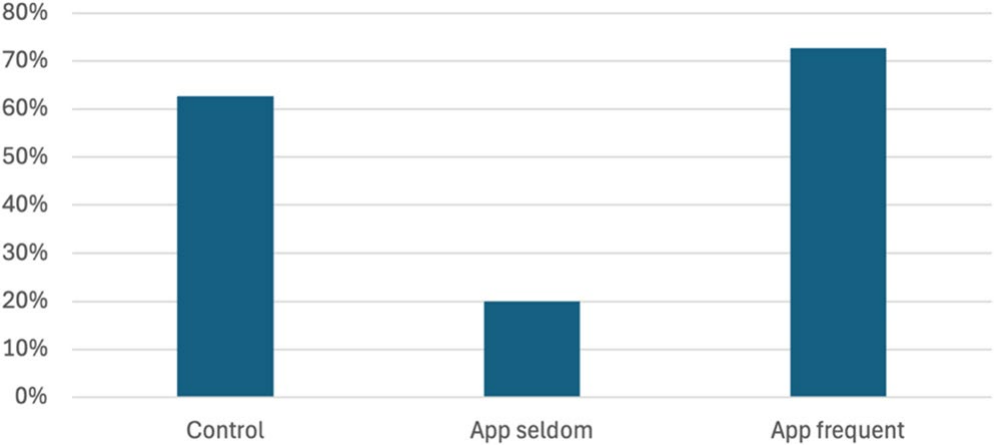
Successful completion of preoperative assessment in different groups

Comparing three groups, a significant difference regarding the rate of successful completion of MTA has been detected (control group 62.6% vs. “app frequent” 72.7% vs. “app seldom” 20.0%, *p* = 0.0039; Fig. 3). Post-hoc comparisons showed no significant difference between control group and “app frequent” group (*p* = 0.1649), whereas both control group and “app frequent” group had a significant higher rate of successful completion of MTA than “app seldom” group (*p* = 0.0152 and *p* = 0.0019, respectively).

### Secondary Outcomes

#### Surgery Rate

Among the 115 participants who successfully completed the preoperative assessment, 106 (92.2%) underwent a planned bariatric surgery. Only 9 (7.8%) patients achieved sufficient weight loss that a bariatric surgery was not indicated anymore. The rate of patients who underwent bariatric surgeries did not differ between control group and app group (56.0% vs. 63.2%, *p* = 0.3296) but differed among the three groups (control group 56.0% vs. “app frequent” 70.1% vs. “app seldom” 10.0%, *p* = 0.0008).

#### Factors Associated with Successful Completion of MTA

In the logistic regression, sex was associated with the possibility of a successful MTA completion: women had approximately twice the chance of successful completion of MTA compared with men, though this result did not reach statistical significance (adjusted odds ratio [aOR] 1.892, 95% CI 0.963–3.716; *p* = 0.0624). Patients who successfully completed the MTA had lower initial weight and BMI (135.4 ± 21.5 kg vs. 144.7 ± 29.2 kg, *p* = 0.0165; 45.8 ±6.0 kg/m^2^ vs. 47.9 ± 7.4 kg/m^2^, *p* = 0.0388). Higher baseline BMI was inversely related to successful completion of MTA (aOR per 1-unit increase 0.946, 95% CI 0.900–0.995; *p* = 0.0301), corresponding to ~ 25% lower odds for every 5 BMI units (0.946⁵≈0.75). Study group allocation was also significantly associated with successful MTA completion (*p* = 0.0131). Compared with the control group, participants in “app frequent” showed a nonsignificant trend toward higher odds of successful completion of MTA (aOR 1.462, 95% CI 0.745–2.869; *p* = 0.2693), whereas “app seldom” had markedly lower odds (aOR 0.124, 95% CI 0.024–0.645; *p* = 0.0131).

#### Weight Loss

Weight loss during MTA was comparable in both control group and app group: control group 4.33 kg vs. app group 3.73 kg; *p* = 0.7564; TWL% control group 3.0% vs. app group 2.7%, *p* = 0.8502.

In the subgroup analysis, the differences were similar for total weight loss (control group 4.33 kg vs. “app seldom” 12.13 kg vs. “app frequent” 2.62 kg, *p* = 0.4191) and for TWL% (control group 3.0% vs. “app seldom” 9.3% vs. and “app frequent” 1.9%, *p* = 0.4732).

In a linear mixed-effects model with fixed effects for group, visit (baseline vs. last), and their interaction, weight decreased significantly from baseline to the last visit across all participants (*p* < 0.0001). Neither the overall differences between groups (*p* = 0.4040) nor the group-by-visit interaction (*p* = 0.0864) were statistically significant, indicating that the magnitude of weight loss was similar across the three groups.

#### Appointment Adherence

Completion rates of required specialist visits were assessed across groups. The number of visits per assessment varied individually: nutritional counseling typically required 5–6 sessions, while psychological consultations and sleep apnea screenings depended on clinical presentation. Assessments were marked as completed only when all necessary visits were finished. Significant differences between groups were found for nutritional counseling, psychological consultation, endocrinology consultation, sleep apnea screening, and upper endoscopy, with the “app frequent” group consistently showing the highest completion rates (Table 2).

**Table 2:** Completion rate of required specialists visits

Specialist	Control	App (total)	App seldom	App frequent	*p*-Value*	*p*-Value**
Nutritional Counseling	70%	83%	33%	89%	**0.0457**	**0.0001**
Psychological Consultation	70%	84%	44%	88%	**0.0414**	**0.0017**
Endocrinology Consultation	73%	84%	33%	89%	0.0862	**0.0001**
Sleep Apnea Screening	77%	84%	44%	88%	0.3005	**0.0045**
Upper Endoscopy	67%	80%	44%	84%	0.0539	**0.0060**

Significant p-values (<0.05) are marked in bold

*Control vs. App (total); **Control vs. App seldom vs. App frequent

#### BIA Measurements

BIA measurement was performed in 177 participants (95.5%) in first and 121 (67.9%) participants in both visits. Between-group comparisons of change from baseline to follow-up showed no significant differences in any BIA component (e.g., fat mass, fat-free mass, total body water, phase angle; Supplementary Table 2).

#### BQL

BQL increased from baseline to follow-up across all groups. Mean (range) change scores (follow-up – baseline) were + 5.11 (− 29.17 to + 44.23) in the control group, + 9.60 (− 13.46 to + 38.46) in “app seldom”, and + 7.08 (− 19.23 to + 55.77) in “app frequent”. A comparison of change scores showed a significant difference (*p* = 0.0010 for control, *p* = 0.0347 for “app seldom”, *p* = 0.0004 for “app frequent”). There were no significant changes in the total score at first visit (*p* = 0.8499), last visit (*p* = 0.1890) and total score changes between the groups (*p* = 0.6028).

#### Assessment of Obesity-Related Medical Conditions

Between baseline and final assessment, the frequency of diagnosed obesity-associated medical conditions increased significantly for insulin resistance (3% first visit vs. 31% last visit, *p* < 0.001), obstructive sleep apnea (32% first visit vs. 48% last visit, *p* < 0.001), continuous positive airway pressure (CPAP) use among patients with sleep apnea (46% first visit vs. 77% last visit, *p* = 0.002), and polycystic ovary syndrome (PCOS) (6% first visit vs. 10% last visit *p* = 0.020), likely reflecting detection of previously undiagnosed conditions during the MTA. Conversely, the reported prevalence of joint pain (67% first visit vs. 49% last visit, *p* < 0.001) and depression (40% first visit vs. 31% last visit, *p* = 0.004) decreased significantly during the study period.

#### Barriers to Successful Completion of MTA

Among the participants, 63 did not complete the MTA. Reported reasons included fear of surgery, unmet expectations regarding the MTA process, lack of health insurance coverage, excessive out-of-pocket costs, insufficient motivation or self-organization, pregnancy, associated medical problems, lack of time, and preference to continue conservative treatment. Seven patients, all in the control group, were unable to complete the MTA due to intercurrent medical or psychological conditions, including stroke, cervical cancer, worsening of pre-existing conditions, systemic lupus erythematosus, depression with alcohol use disorder, surgical complications advised against externally, and uterine fibroids requiring hysterectomy. In all cases, the decision not to proceed was made by the patients or external specialists, not by our bariatric team.

## Discussion

In this pilot trial, we investigated the feasibility and effectiveness of using a mobile app as a supportive tool for the preoperative assessment prior to bariatric surgery. Our results clearly demonstrated that a frequent usage of the app increased the possibility to complete the preoperative assessment successfully and led to a higher rate to receive bariatric surgery.

The overall rate of successful completion of MTA of 64.6% in completing the multidisciplinary preoperative assessment aligns with previously reported attrition rates before bariatric surgery, where dropout rates of 30–60% were observed [32, 33]. The intention-to-treat analysis showed no significant difference between the app and control groups when analyzed strictly by randomized allocation. This is in line with the results of a previous 8-week randomized trial of a digital health intervention for bariatric surgery candidates, where changes in BMI were small and no statistical difference was found between groups [34]. Similarly, a randomized study assessing the impact of adding an application to the nutritional preparation process on preoperative nutritional knowledge, physical, and behavioral parameters among candidates reported that the app mainly affected physical activity initiation, without consistent additional advantages across other preoperative outcomes [35]. 

However, the mentioned studies set different foci and endpoints than ours and did not investigate the impact of the actual app engagement on success rate of completing the multidisciplinary preoperative assessment. Participants categorized as frequent users (≥ 34% of recommended content usage) had substantially higher successful MTA completion and surgery rates than seldom users and standard care in post hoc comparisons. This result suggests that the app per se is not beneficial or harmful, but its effectiveness is contingent upon meaningful user engagement. The heterogeneity of app engagement was also described in the 8-week digital intervention trial in bariatric surgery candidates [34], showing that a significant portion of participants underuse the digital tools. However, our engagement-stratified findings should be interpreted with caution, as higher engagement may reflect underlying patient characteristics, such as motivation, health literacy, or readiness for lifestyle change, rather than a direct causal effect of the app. Conversely, low engagement may serve as a marker of barriers that also reduce the likelihood of successful MTA completion. In this regard, the app could function not only as an adherence-enhancing tool but also as a risk stratification instrument, where early low engagement flags patients who may benefit from additional human support, such as navigator outreach and targeted barrier resolution.

In our cohort, most frequent barriers to a successful MTA completion included fear of complications, misaligned expectations about the assessment process, financial constraints (inadequate insurance coverage or high costs), motivational difficulties, poor self-organization, time limitations, and preference for conservative management. These findings are consistent with previous investigations identifying factors among those who discontinued the process after initial clinic contact [16, 36]. A qualitative study in New Zealand emphasizes that “structural” barriers, such as practical support, navigating the medical system and health literacy, could affect preoperative attrition rates, even when the preoperative assessment and surgery are covered by most health insurances, as in Germany [37]. Notably, all seven patients who could not complete the MTA due to intercurrent medical conditions were in the control group. While this may partly reflect chance, the absence of close digital communication in the control group could have contributed, as the app’s automated alerts for missed appointments may facilitate earlier identification of patients facing medical or psychosocial challenges and enable timely support by the bariatric team.

Our finding that female sex was independently associated with successful completion is consistent with prior literature showing that male sex is a risk factor for dropout before surgery [38]. The inverse relationship between baseline BMI and the rate of completion of MTA suggests that patients with more severe obesity face greater barriers, potentially including more frequent obesity-associated medical conditions requiring management, or increased logistical challenges in attending multiple appointments. These patients may benefit from more intensive support or modified assessment pathways.

Preoperative weight loss is common during MTA [39, 40]. In our study cohort, patients achieved modest weight loss with mean reductions of 4.33 kg (3.0% TWL) in the control group and 3.73 kg (2.7% TWL) in the app group, with no significant difference between groups. This is expected, as weight loss during the MTA is primarily driven by dietary counseling, lifestyle changes, and management of obesity-associated medical problems rather than the app itself. The app was designed to facilitate adherence to scheduled appointments and assessments, not to serve as a weight loss intervention. Therefore, no conclusions regarding the effectiveness of digital interventions for weight loss can be drawn from this study. Notably, in Germany, health insurance requires patients to demonstrate a documented attempt at conservative weight management as part of the MTA. If patients complete the MTA without achieving significant weight loss through these conservative measures, they could qualify for insurance-covered MBS. The improvement in quality-of-life scores across all groups, despite minimal weight change, likely reflects the positive psychological impact of entering a structured treatment pathway and the anticipation of surgical intervention [34]. 

The significant increase in diagnosed obesity-associated medical problems such as insulin resistance, obstructive sleep apnea, and PCOS during the assessment period underscores the diagnostic value of the MTA. These findings likely reflect previously undiagnosed conditions that were identified through systematic evaluation rather than new onset of disease and support the importance of a thorough multidisciplinary workup prior to MBS.

The smartphone application used in this study was specifically designed to guide patients through standardized postoperative assessments. We did not evaluate alternative applications, as no comparable tool meeting these requirements was available at the time of the study. However, the observed effects are likely attributable to the structured self-assessment approach itself rather than to the specific application, and we anticipate that any similarly designed tool would achieve comparable results.

Some limitations of our study must be acknowledged. First, the relatively low enrollment rate (24.7% of screened patients) might have introduced selection bias. Patients who perceived limited personal benefit from digital tools may have declined participation, potentially excluding individuals who might have responded well to structured digital support. On the other side, those lacking motivation for structured self-management may have declined to participate in the study, despite potentially benefiting most from the app’s reminder and accountability features. This selection effect limits our ability to generalize findings to the broader population of bariatric surgery candidates, especially those at highest risk of attrition. Second, the exploratory nature of the study and modest sample size limited statistical power for detecting small to moderate effects in the intention-to-treat analysis. Third, the post hoc stratification by engagement introduces the risk of selection bias. Participants with higher engagement may have differed in unmeasured cofactors that independently predicted completion of MTA. Importantly, our data do not allow us to determine whether low-engagement users were consistently disengaged from the start or whether their engagement declined at a specific point during the MTA. It is plausible that some low-engagement users had silently decided not to pursue surgery without formally withdrawing, which would explain both their low app use and non-completion of the MTA. Fourth, our study was conducted at a single tertiary bariatric center in a German-speaking region, which may limit generalizability to other healthcare settings, languages, or cultural contexts. Future studies should incorporate more comprehensive psychological evaluation to identify barriers to sustained app engagement and resolve factors associated with high or low engagement. This could help with prognosis whether specific baseline characteristics or behavioral patterns can predict if participants belong to the low engagement category. Long-term follow-up data could provide insights whether preoperative engagement predicts postoperative long-term outcomes, including weight loss, development of complications and risk of weight regain.

## Conclusion

Our results suggest that digital health tools can support patients during the preoperative bariatric surgery pathway, but their effectiveness depends on adequate user engagement. Higher app engagement was associated with higher successful MTA completion rates, while low engagement may indicate patients who require additional support. Future studies should investigate strategies to promote sustained engagement and explore whether early identification of low-engagement patients can improve outcomes.

## Supplementary Information

Below is the link to the electronic supplementary material.

Supplementary Material 1 (DOCX 30.7 KB)

## Data Availability

The datasets generated and analyzed during the current study are available from the corresponding author upon reasonable request.
