# Supplementary material for: Preoperative Use of a Mobile Application Within the Multidisciplinary Team Approach – A Randomized Controlled Clinical Trial
Source: Obes Surg. 2026 Jun 15;36(8):4084–95. doi: 10.1007/s11695-026-08768-1 (PMC13429525; doi:10.1007/s11695-026-08768-1)
Supplement: Supplementary file 1 — Supplementary Material 1 (DOCX 30.7 KB) [file 11695_2026_8768_MOESM1_ESM.docx]

**Supplementary materials**

Supplementary figure 1: ROC-Curve for Cutoff App Use. Red Dot symbolizes app using rate of 34%

Supplementary table 1: App questionnaire

| **Questions** | **Answer Options** |
| --- | --- |
| **SECTION: Nutritional Counseling** |  |
| Meeting with your nutritionist is important for your surgery preparation. How many appointments will you have with your nutritionist? | Select rolling number 3–6 |
| Have you had a nutritional counseling appointment in the last 7 days? | Yes / No |
| Please select the reason for not attending your appointment: | There was none scheduled this week I have not had time I am struggling mentally or having doubts Finding it difficult to contact the right person Other |
| **SECTION: Physical Activity** |  |
| Sports Diary: Did you engage in physical activity today? | Yes No I am unable to exercise due to medical reasons (confirmed by a doctor's note) |
| What type of physical activity did you do? | Multiple choice: Running / Hiking Jogging Cycling Swimming Nordic walking Fitness Weightlifting Aerobics / Gymnastics / Pilates Other sport |
| How long did you exercise for? | Scale: 10 min to ≥180 min (VAS) |
| How many steps did you take today? | Scale: 0 to 10,000 in steps of 100 (VAS) |
| **SECTION: Dietary Self-Monitoring** |  |
| How many calories did you consume today? | Scale: 0 to 6,000 kcal (VAS) in steps of 100 |
| **SECTION: Weight Monitoring** |  |
| What is your weight? | Scale: 50 to 250 kg (picker) |
| **SECTION: General Information** |  |
| If you would like more information about bariatric surgery, tap the "Info" button on the top right of this screen. | OK |
| Have you scheduled an appointment at a practice specializing in obesity? This appointment is not mandatory but strongly recommended. | Yes No I did not intend to make an appointment |
| **SECTION: Specialist Appointments** |  |
| The following questions refer to the specialist appointments required as part of the multidisciplinary assessment. Please note: Mandatory appointments: endocrinologist, psychologist, ENT (sleep apnea diagnosis), gastroenterologist (gastroscopy). Optional appointments: Orthopedist (for joint problems limiting physical activity), cardiologist or pulmonologist (only for pre-existing cardiac or pulmonary conditions). pH-metry is only required for reflux symptoms. | OK |
| Which of the following specialist appointments are required as part of your multidisciplinary assessment? | Multi-select: Endocrinologist Orthopedist Psychologist / Neurologist / Psychiatrist Gastroenterologist / Endoscopy Ear, Nose & Throat (ENT/GP) Cardiologist |
| **SECTION: Final Appointments** |  |
| Have you had your final appointment with your endocrinologist? | Yes / No |
| Have you had your final appointment with your orthopedist? | Yes No I do not have any back, knee, or hip pain |
| Have you had your final appointment for the psychological assessment? | Yes / No |
| Have you had your final appointment with your gastroenterologist / endoscopy? | Yes / No |
| Have you had your final appointment with ear, nose & throat (ENT/GP)? | Yes / No |
| Have you had your final appointment with your cardiologist? | Yes / No |
| **SECTION: Surgery Preparation** |  |
| Do you have an appointment for surgery preparation? | Yes No |
| Do you have any of the following conditions? | Multi-select: Knee / Back / Hip pain heart disease Reflux (heartburn) Abnormal findings on cardiorespiratory polygraphy |
| Have you collected the following documents from your appointments? | Multi-select: Nutritional counseling Sports diary Psychologist Endocrinologist ENT Sleep lab Gastroscopy pH-metry (in case of reflux symptoms) Orthopedist Cardiologist Specialist practice for obesity (not mandatory) |
| Please bring all documents from the following appointments to your surgery planning visit: nutritional counseling, endocrinologist, psychologist, gastroenterologist/endoscopy (gastroscopy), sports diary, polygraphy (sleep apnea diagnosis by ENT, GP, or lung specialist). If these documents are missing, you cannot be scheduled for surgery. If you have other documents from orthopedists, cardiologists, and pH-metry, please bring them with you. | OK |

Supplementary table 2: BIA measurements

| BIA-Parameter | Control group | | App group | | p-value |
| --- | --- | --- | --- | --- | --- |
|  | First Visit | Last Visit | First Visit | Last Visit |  |
| resting metabolic rate in kcal | 1828.1±292.7 | 1823.1±296.6 | 1785.2±273.1 | 1799.1±278.0 | 0.4553 |
| phase angle in ° | 6.0±0.7 | 6.0±0.7 | 6.0±0.7 | 6.0±0.8 | 0.3133 |
| total body water in l | 54.2±11.7 | 54.2±12.1 | 52.4±11.1 | 52.4±11.1 | 0.8764 |
| lean body mass in kg | 74.1±16.0 | 74.0±16.6 | 71.5±15.2 | 71.4±15.5 | 0.8253 |
| extra cellular mass (ECM) in kg | 35.8±7.6 | 35.8±8.1 | 34.6±7.4 | 34.2±7.5 | 0.6374 |
| body cell mass (BCM) in kg | 38.3±9.2 | 38.2±9.4 | 37.0±8.6 | 37.2±8.9 | 0.6816 |
| ECM/BCM | 0.9±0.1 | 0.9±0.1 | 0.9±0.1 | 0.9±0.1 | 0.3159 |
| cell mass in % | 51.6±3.5 | 51.5±3.5 | 51.6±3.2 | 51.9±3.6 | 0.3149 |
| Body Fat in kg | 67.0±16.0 | 65.8±17.4 | 62.5±14.5 | 61.6±14.8 | 0.9793 |
| Body Fat in % | 47.4±6.8 | 46.8±6.9 | 46.7±7.6 | 46.3±7.9 | 0.9565 |

BIA, body impedance analysis.
